# Supplementary material for: The Use of Molecular Dynamics Simulation Method to Quantitatively Evaluate the Affinity between HBV Antigen T Cell Epitope Peptides and HLA-A Molecules
Source: Int J Mol Sci. 2022 Apr 22;23(9):4629. doi: 10.3390/ijms23094629 (PMC9105472; doi:10.3390/ijms23094629)
Supplement: Supplementary file 1 [file ijms-23-04629-s001.zip › TableS1.pdf]

Table S1. Test parameters of the modelled structures

Modelled structure	Test parameters		
	Alpha Chain	Beta Chain	Heterodimer
HLA-A*11:02	Template:5WJL<br>Template Resolution:3.15Å<br>Template Allele: HLA-A*11:01<br>Identity:0.99<br>Verify Score:0.9891<br>Verify Score:87.0079<br>Core:0.92<br>Allow:0.076<br>Gener:0<br>Disall:0.004	Template:5WJL	Ligand RMSD:0.977<br>Clashscore:2.31<br>Poor rotamers:3.37%<br>Ramachandran favored:97.30%<br>Molprobity score:1.54<br>Total RMSD:0.005
HLA-A*26:01	Template:6AT9<br>Template Resolution:2.95 Å<br>Template Allele: HLA-A*01:01<br>Identity:0.91<br>Verify Score:0.9891<br>Verify Score:80.8765<br>Core:0.88<br>Allow:0.116<br>Gener:0.004<br>Disall:0	Template:6AT9	Ligand RMSD:0.796<br>Clashscore:4.86<br>Poor rotamers:1.22%<br>Ramachandran favored:96.76%<br>Molprobity score:1.52<br>Total RMSD:0.113
HLA-A*33:03	Template:5WJL<br>Template Resolution:3.15 Å<br>Template Allele: HLA-A*11:01<br>Identity:0.93<br>Verify Score:0.9635<br>Verify Score:92.8571<br>Core:0.917<br>Allow:0.079<br>Gener:0<br>Disall:0.004	Template:5WJL	Ligand RMSD:0.981<br>Clashscore:2.55<br>Poor rotamers:3.67%<br>Ramachandran favored:97.30%<br>Molprobity score:1.6<br>Total RMSD:0.047
HLA-A*31:01	Template:5WJL<br>Template Resolution:3.15 Å<br>Template Allele: HLA-A*11:01<br>Identity:0.93<br>Verify Score:0.9599<br>Verify Score:92.4603<br>Core:0.917<br>Allow:0.079<br>Gener:0<br>Disall:0.004	Template:5WJL	Ligand RMSD:0.397<br>Clashscore:2.78<br>Poor rotamers:3.66%<br>Ramachandran favored:97.30%<br>Molprobity score:1.63<br>Total RMSD:0.413
